# CD61 promotes the differentiation of canine ADMSCs into PGC-like cells through modulation of TGF-β signaling

**DOI:** 10.1038/srep43851

**Published:** 2017-03-03

**Authors:** Jia Fang, Yudong Wei, Changrong Lv, Sha Peng, Shanting Zhao, Jinlian Hua

**Affiliations:** 1 College of Veterinary Medicine, Shaanxi Centre of Stem Cells Engineering & Technology, Northwest A&F University, Yangling, Shaanxi, 712100, China

## Abstract

Previous studies have shown that CD61 (integrin-β3) promotes the differentiation of human umbilical cord mesenchymal stem cells (hUC-MSCs) into germ-like cells. However, the mechanism remains unclear. In this study, we showed that overexpression of CD61 in canine adipose-derived mesenchymal stem cells (cADMSCs) promotes their differentiation into primordial germ cell (PGC)-like cells. Quantitative real-time PCR, immunocytochemistry and western blot detected higher levels of PGC-specific markers in CD61-overexpressed cADMSCs compared with those in control cells. Moreover, phosphorylation of Smad2, a downstream mediator of transforming growth factor beta (TGF-β), was increased in CD61-overexpressed cADMSCs than that in control cells. However, the expression of PGC-specific markers was downregulated in cADMSCs treated with a TGF-β inhibitor. These results suggested that CD61 could induce cADMSCs to differentiate into PGC-like cells by relying on the activation of TGF-β pathway. ADMSCs possess a considerable potential in treating the infertility of rare animal species.

Mesenchymal stem cells (MSCs) are multipotent stem cells that can differentiate into various cell types including osteoblasts, chondrocytes, adipocytes, beta-pancreatic islets cells, cardiac cells and neuronal cells *in vitro* or *in vivo*^1^. MSCs can be obtained from different adult tissues such as bone marrow^2^, adipose tissue^3^, umbilical blood^4^, umbilical cord^5^ and peripheral blood^6^. Adipose-derived mesenchymal stem cells (ADMSCs) have one big advantage over other tissue-derived MSCs in that they are easily accessible. Recently, MSC therapy is an exceptionally promising method in the veterinary field because of its good curative effects^7^.

CD61, also named integrin-β3, is an adhesion molecule that is widely distributed on cell surface. This molecule regulates extracellular signals and intracellular pathways and modulates the physiology of the body. CD61 also plays a key role in embryonic development^8^ and sperm maturation^9^.

Some severe injuries and acquired diseases affect canine’s health and cause obstacles for breeding. Induction of stem cells to differentiate into germ cells (GCs) makes breeding possible. It has been reported that MSCs derived from bone marrow and umbilical cord can differentiate into germ-like cells^10^,^11^. Our previous investigation also demonstrated that human umbilical cord mesenchymal stem cells (hUC-MSCs) can potentially differentiate into germ-like cells under treatment with CD61 and bone morphogenetic protein 4 (BMP4)^12^. However, the mechanisms remain unclear.

In the present study, human CD61 was overexpressed in canine adipose-derived mesenchymal stem cells (cADMSCs). We investigated the effects of CD61 on the induction of cADMSCs to differentiate into primordial germ cell (PGC)-like cells and the possible mechanism. Results showed that integrin-β3-transforming growth factor beta (TGF-β) pathway may play a key role in the differentiation of male germ-like cells derived from cADMSCs.

## Results

### CD61 expression was increased in CD61-overexpressed cADMSCs

**Figure 1:**
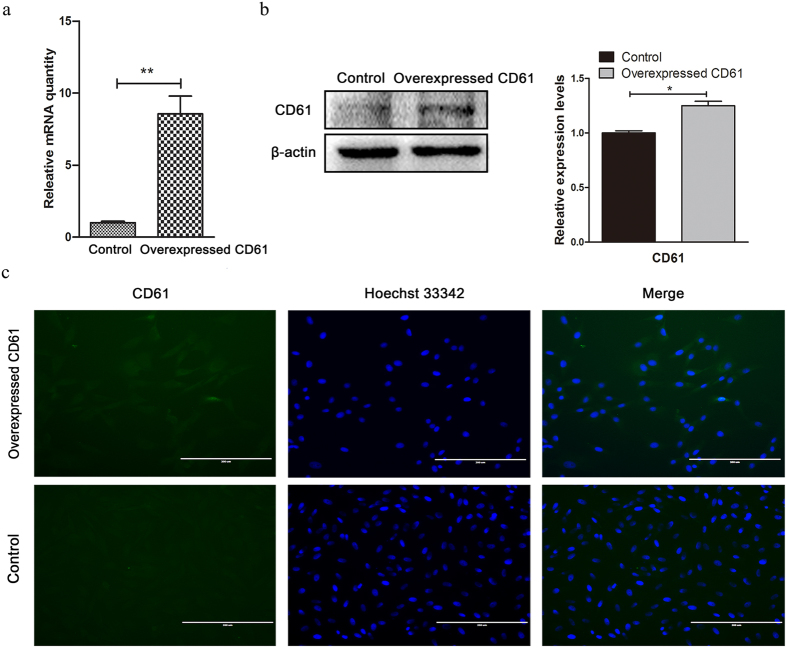
CD61 expression of control cells and CD61-overexpressed cells. mRNA and protein expression levels of CD61 in control cells and CD61-overexpressed cells analyzed by (**a**) quantitative real-time PCR (QRT-PCR), **P < 0.01, (**b**) western blot, *P < 0.05, and (**c**) immunocytochemistry. Bar = 200 μm.

CD61 was overexpressed by the third passage of cADMSCs following transfection plasmid with pcDNA3.1-beta-3. The pcDNA3.1 (+) was used as a control plasmid using the same conditions. cADMSCs were collected at 48 h after transfection. According to quantitative real-time PCR (QRT-PCR) (Fig. 1a), Western blot (P < 0.05) (Fig. 1b) and immunocytochemistry (Fig. 1c), the expression level of CD61 in cADMSCs transfected with pcDNA3.1-beta-3 was significantly higher than that in control cells.

### CD61 promoted cADMSCs to differentiate into PGC-like cells

**Figure 2:**
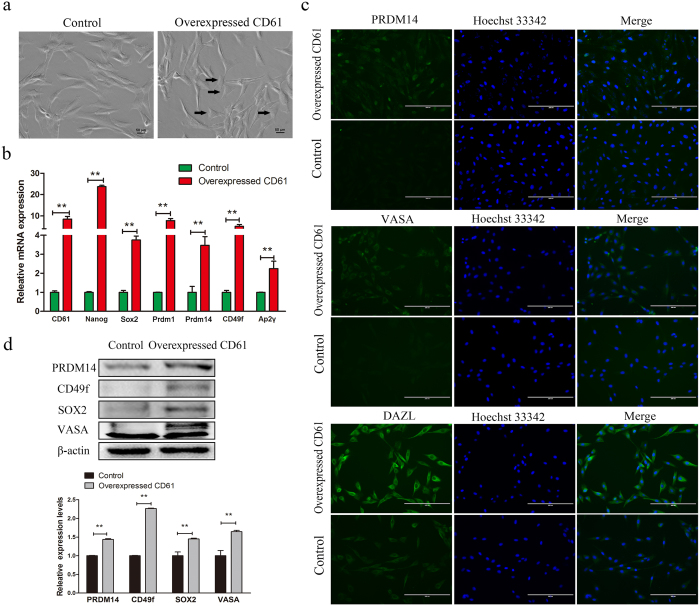
Morphology and expression of primordial germ cell (PGC)-related markers in CD61- overexpressed cells and control cells. (**a**) The morphology of CD61-overexpressed cells and control cells. Canine adipose-derived mesenchymal stem cells (cADMSCs) with overexpression of CD61 became short spindle-shaped or triangular. (**b**) Gene expression (*CD61, Nanog, SOX2, Prdm1, Prdm14, CD49f* and *Ap2γ*) of control and CD61- overexpressed cells was detected by QRT-PCR. **P < 0.01. (**c**) Immunocytochemistry of PRDM14, VASA and DAZL in CD61-overexpressed cADMSCs and control cells. Bar = 200 μm. (**d**) Western blot of PGC specific markers (PRDM14, CD49f, SOX2 and VASA) in CD61-overexpressed cells and control cells. **P < 0.01.

A few numbers of CD61-overexpressed cADMSCs became short-spindled (Fig. 2a) at 48 h after transfection. Some PGC-specific markers including *Prdm1, Prdm14, AP2γ, CD49f*, and stem cell markers such as *Nanog* and *Sox2*, were expressed at higher levels in CD61-overexpressed cADMSCs compared with those in controls, as shown by QRT-PCR (P < 0.01) (Fig. 2b). The levels of PRDM14, VASA and DAZL were also increased in CD61-overexpressed cADMSCs compared with those in control cells, as detected by immunocytochemistry (Fig. 2c). PRDM14, CD49f, SOX2 and VASA were increased in CD61-overexpressed cADMSCs compared with those in control cells, as examined by western blot (P < 0.01) (Fig. 2d). In addition, the levels of meiotic markers, including STRA8 (P < 0.01) and SYCP3 (P < 0.05), were increased in CD61-overexpressed cADMSCs compared with those in control cells, as detected by western blot (Fig. S1a).

### TGF-β signaling was activated in CD61-overexpressed cADMSCs

**Figure 3:**
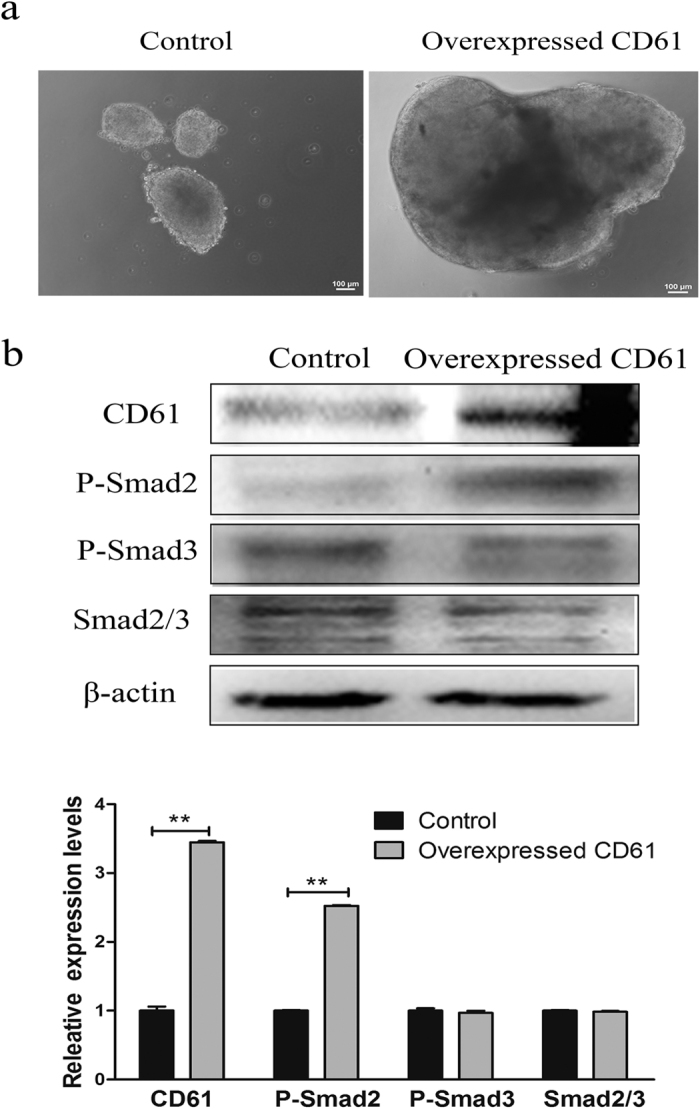
Activation of the transforming growth factor beta (TGF-β) pathway in CD61-overexpressed cADMSCs. (**a**) EB formation in control and CD61-overexpressed cells. (**b**) Western blot of TGF-β markers (p-Smad2, p-Smad3 and Smad2/3) in CD61-overexpressed cells and control cells. **P < 0.01.

A part of cADMSCs was cultured in suspension for 16 h after transfection. Cells grew closely together and cADMSCs transfected with CD61 formed larger cell clusters than those of control cells (Fig. 3a). TGF-β signaling plays a critical role in regulating the pluripotency of embryonic stem cells. Thus, we investigated the levels of TGF-β downstream mediators using western blot (Fig. 3b). The phosphorylation level of Smad2 (p-Smad2) was increased in CD61-overexpressed cADMSCs compared with that in the control group (P < 0.01). However, no significant difference was detected in Smad2/3 and the phosphorylation level of Smad3 (p-Smad3).

### TGF-β inhibitor suppressed the expression of PGC-specific markers

**Figure 4:**
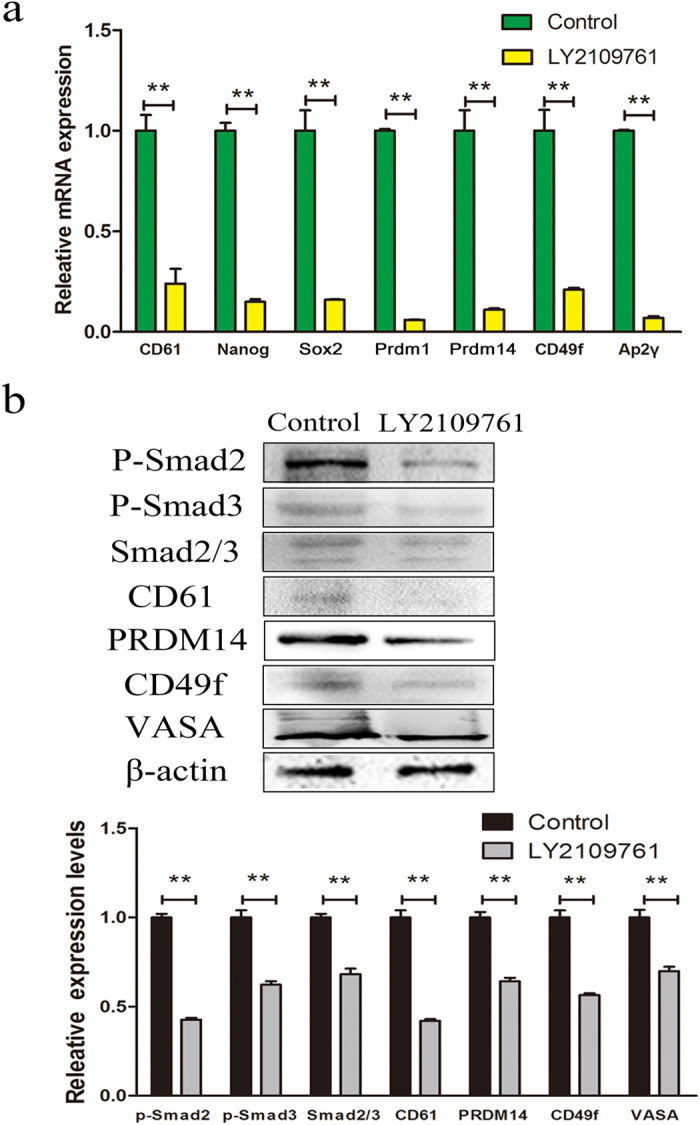
Expression of PGC-related markers in LY2109761-treated cells and control cells. (**a**) PGC-related markers *CD61, Prdm1, Prdm14, CD49f, Ap2γ* and stem cell markers *Nanog* and *Sox2* were examined by QRT-PCR in LY2109761-treated cells and control cells. **P < 0.01. (**b**) Phosphorylation of Smad2, Smad3, total Smad2/3 and PGC-related markers *CD61, PRDM14, CD49f* and VASA was examined in LY2109761-treated cells and control cells by western blot. *t*-tests **P < 0.01.

To examine the functional relevance of the TGF-β pathway in PGC-specific differentiation of CD61-transfected cADMSCs, we treated cADMSCs with 0.01 mM LY2109761, a TGF-β inhibitor, for two days. PGC-specific and stem cell markers were examined by QRT-PCR (Fig. 4a) and western blot (Fig. 4b). The results showed that the relative expression of *CD61, Nanog, Sox2, Prdm1, Prdm14, CD49f* and *AP2γ* decreased in mRNA level in LY2109761-treated cells compared with that in the control (P < 0.01). The levels of meiotic markers including STRA8 and SYCP3, were decreased in LY2109761-treated cADMSCs compared with those in control cells, as detected by western blot (P < 0.01) (Fig. S1b). Moreover, the levels of Smad2/3, CD61, PRDM14, CD49f and VASA and the phosphorylation of Smad2 and Smad3 were decreased in LY2109761-treated group compared with those in the control (P < 0.01).

### TGF-β1 promoted the expression of PGC-specific markers

**Figure 5:**
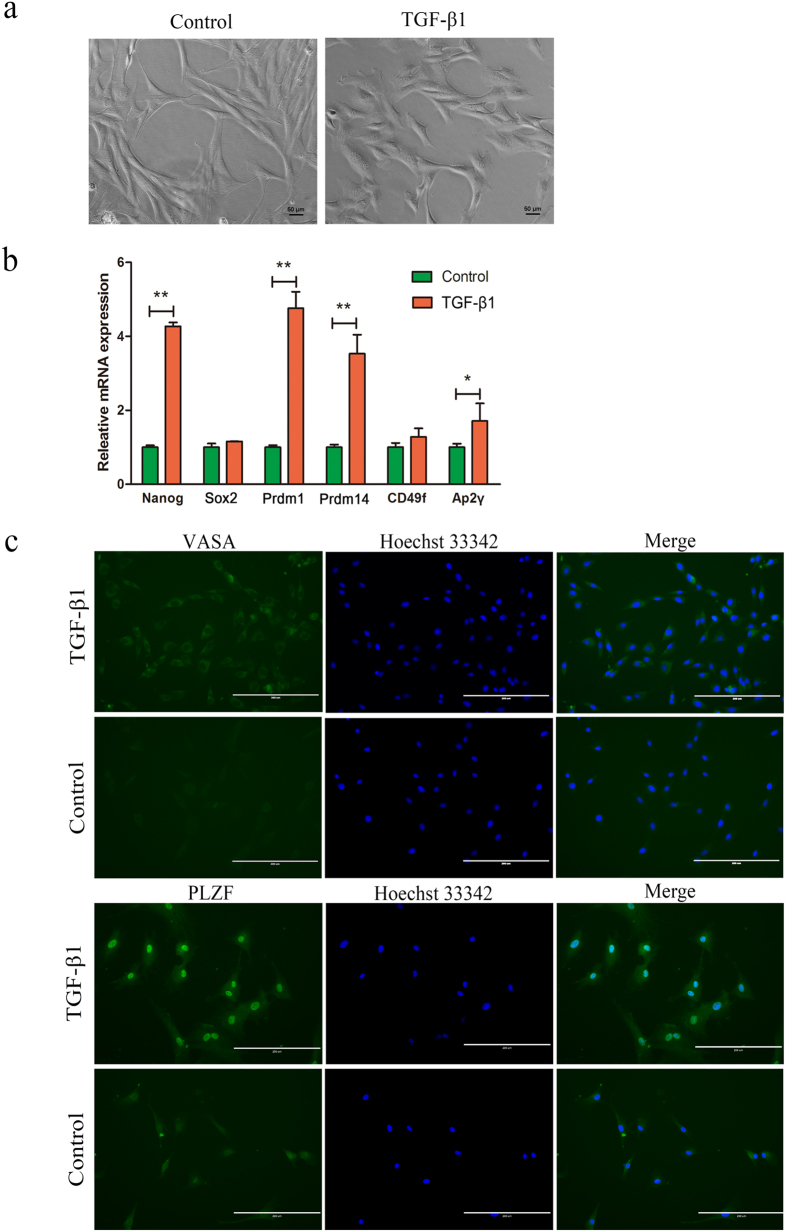
Expression of PGC-related markers in TGF-β1 treated cells and control cells. (**a**) Morphology of TGF-β1 treated cells and control cells. cADMSCs became flat, short-spindled and triangular or polygon-shaped after TGF-β1 treatment. (**b**) Gene expression (*Prdm1, Prdm14, CD49f, Ap2γ* and *Nanog*) of CD61-overexpressed cells and control cells analyzed by QRT-PCR. **P < 0.01; *P < 0.05. (**c**) Immunocytochemistry of PGC-related markers (VASA and PLZF) in CD61-overexpressed and control cells. Bar = 200 μm.

To confirm the effect of TGF-β pathway during cADMSCs differentiation into PGC-like cells, we treated cADMSCs with 10 ng/ml TGF-β1 for 10 days. The cADMSCs became flat, short-spindled and triangle or polygon-shaped (Fig. 5a) after the treatment. PGC-specific and stem cell markers were examined by QRT-PCR (Fig. 5b) and immunocytochemistry (Fig. 5c). The mRNA relative expression levels of *Nanog, Prdm1, Prdm14* (P < 0.01) and *AP2γ* (P < 0.05) were increased in TGF-β1 treated cADMSCs compared with those in control. Glyceraldehyde 3-phosphate dehydrogenase (*Gapdh*) was used as the control house-keeping gene. The protein expression levels of VASA and PLZF were increased in TGF-β1 treated cADMSCs compared with those in control cells. The levels of meiotic markers, including STRA8 (P < 0.01) and SYCP3 (P < 0.05), were increased in TGF-β1-treated cADMSCs compared with those in control cells, as detected by western blot (Fig. S1c).

## Discussion

ADMSCs are regarded the ideal candidates for stem cell therapy and effective in many disease models^13^. Compared with embryonic stem cells, ADMSCs are non-immunogenic, non-oncogenic and easily accessible. ADMSCs, including adipocytes, chondrocytes, muscle cells and germ-like cells, exhibit multiple differentiation capabilities^14^,^15^,^16^,^17^. ADMSCs possess a considerable potential in treating the infertility of rare animal species.

CD61 is involved in cell adhesion and cell surface-mediated signaling. In our experiments, cADMSCs transfected with CD61 formed larger cell clusters than those of control cells, thereby suggesting that CD61 may play a role in cADMSCs’ adhesion and survival^18^. CD61 also plays an important role in PGC development. PGC-like cells, which are positive for SSEA1 and CD61, can develop into spermatid cells^19^. Our previous studies showed that CD61 promotes hUC-MSCs to differentiate into male germ-like cells^12^. Nevertheless, the mechanism of CD61 in PGC development remains unclear. In our present experiments, overexpression of CD61 in cADMSCs could upregulate the expression of PGC-specific markers, including PRDM1, PRDM14, AP2γ, CD49f, SOX2 and Nanog. Immunocytochemistry result showed that labelling of DAZL and VASA in CD61-overexpressed cADMSCs was more frequent compared with that in control cells. This result confirmed that CD61 may play a role in PGC development. Medrano^20^ proved that six germ line-related factors (PRDM1, PRDM14, LIN28A, DAZL, VASA and SYCP3) induce human somatic cells to display meiotic GC-like features; furthermore, only approximately 1% of these cells show complete meiosis, as demonstrated by their haploid status and the expression of several post-meiotic markers. We also demonstrated that CD61 could promote cADMSCs to differentiate into PGC-like cells. PGC-specific markers, including Prdm1, Prdm14, AP2γ, CD49f, SOX2 and Nanog, were increased by overexpressed CD61.

**Figure 6:**
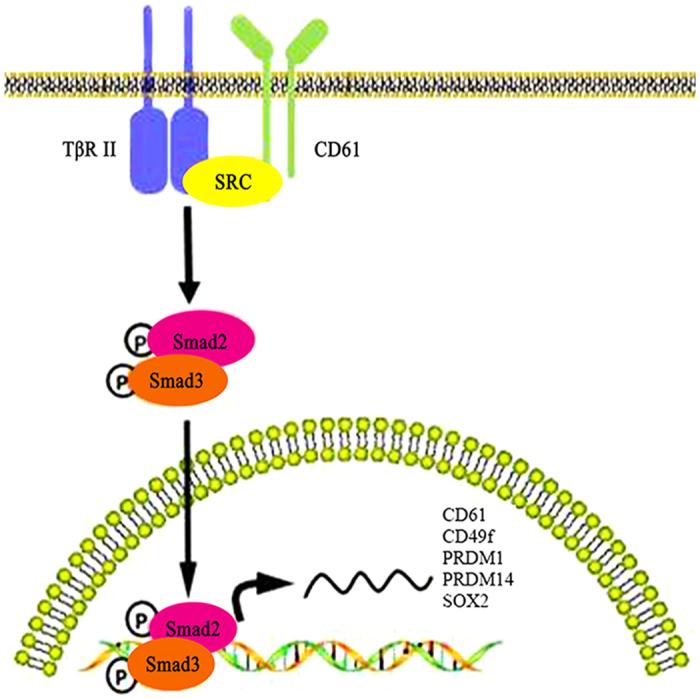
CD61 played a role in induction of PGC differentiation by activating TGF-β signaling pathway. After overexpression of αvβ3 integrin, CD61 interacted physically with TβR-II, thereby leading to its phosphorylation by Src. The activation of TβR-II phosphorylated and promoted Smad2/3 transportation to the nucleus. Phosphorylated Smad2/3 combined with the promoters of several differentiation-related genes, such as CD61, CD49f, PRDM1, PRDM14 and SOX2, which resulted in the regulation of their expression.

Previous studies have shown that MSCs can differentiate into GCs by several different methods. Retinoic acid (RA) plays an important role in the proliferation and differentiation of PGCs. RA promotes the differentiation of bone marrow MSCs and skin-derived stem cells into male GCs *in vitro*^21^. The TGF-β pathway is considerably important for controlling cell proliferation and differentiation. TGF-β signal is present in foetal and neonatal developing testis and required for GC development^22^,^23^. BMP4, a member of the TGF-β superfamily, is essential for the PGC formation from initially sexually indifferent germline cells^24^. BMP4 promotes iPS cell differentiation into male GCs^25^. It also can successfully induce MSCs to differentiate into PGCs and further increase the levels of male germ-cell markers, such as STRA8, SCP3, DMRT1 and PLZF^26^. Activin, another TGF-β family member, performs important roles in migration, survival and proliferation of PGCs^19^. Activin A could induce mouse skin-derived stem cells to differentiate into PGCs and affect meiotic initiation^27^. A comparison experiment demonstrated that the differentiation efficiency of MSCs by TGF-β1 is higher than that by RA^28^. In our studies, similar to the overexpression of CD61, TGF-β1 can promote cADMSCs to differentiate into PGC-like cells. CD61 interacts physically with the TGF-β receptor type II (TβRII) and phosphorylates by Src during breast cancer growth and metastasis^29^. In our studies, CD61 could induce cADMSCs to differentiate into PGCs-like cells by activating the TGF-β pathway. On the contrary, the expression of PGC markers could be reduced by adding TGF-β receptor inhibitor LY2109761. The stem cell markers SOX2 and Nanog were also decreased by inhibiting TGF-β signal, which indicated that the TGF-β signaling regulates the differentiation and development of PGCs. Interestingly, the expression level of CD61 was also decreased by inhibiting TGF-β signal, thereby suggesting the autoregulation interaction of CD61 and TGF-β signaling. We hypothesised that CD61 physically interacted with the TβR-II and consequently promoted the Smad2/3 transportation into the nucleus. Phosphorylated Smad2/3 combined with the promoters of several differentiation-related genes, such as *CD61, CD49f, Prdm1, Prdm14* and *Sox2*, and regulated their expression (Fig. 6).

Our work demonstrated that CD61-positive cADMSCs can differentiate into PGC-like cells. Moreover, CD61 plays a role in inducing PGC differentiation by activating the TGF-β signal pathway.

## Methods

### Cell isolation, identification and culture

Canine adipose tissue was harvested from abdominal subcutaneous fat from three male beagle canine after anaesthesia by zoletil (Virbac group, France) injection. The canine was cared for in Experimental Animal Center of Northwest A&F University. The experiment was approved by the committee of Shaanxi Centre of Stem Cells Engineering & Technology, Northwest A&F University. The canine was used according to Chinese Laboratory Animal Guidelines.

The isolation and identification of cADMSCs were described previously^16^. Briefly, adipose tissue was minced and digested by collagenase type I solution (Roche Diagnostics, Switzerland). The cells were identified using surface markers by flow cytometry and *in vitro*-induced differentiation. The isolated cADMSCs are positive for CD73, CD105 (Fig. S2), CD44, CD90 and CD166, whereas negative for CD34 and CD45; these cells could also differentiate into adipocytes, osteoblasts and chondrocytes under induction conditions^16^.

The cADMSCs were cultured in cell culture dish in normal culture medium which contained α-MEM (Invitrogen, Carlsbad, CA) supplemented with 10% FBS (HyClone, UT, USA), 2 mM L-glutamine and 1% non-essential amino acids (Invitrogen), in a humid atmosphere with 5% CO_2_ at 37 °C. Cells were dissociated every 2 days with trypsin-EDTA (Invitrogen). For all experimental set-ups, cells were used between passages 2 to passage 4.

### Cell transfection

The plasmids pcDNA3.1-beta-3(Addgene, Cambridge, USA) and pcDNA3.1 (+) were transfected by Turbofect (Thermo Scientific, NH, USA) according to the manufacturer’s recommendations. The cells were plated at a density of 1 × 10^5^ cells per mL with normal culture medium in 6-well plates in preparation for transfection. Eight hours after transfection, the medium was discarded and replaced with normal culture medium and incubated for another 48 h.

### Embryoid Body (EB) Formation

The induction protocol was referred as Li^22^. In briefly, 2 × 10^5^ cells were seeded into 35-mm suspension culture plates with 1.5 ml normal culture medium. EBs were formed at 16 h after suspension cultivation.

### QRT-PCR analysis

The total RNA of cADMSCs was extracted by using Trizol reagent (Takara, Japan) according to the manufacturer’s instructions. Reverse Transcriptase Reagent kit (Thermo Scientific) was used to reverse transcript RNA into cDNA according to the manufacturer’s instructions. QRT-PCR was performed in the CFX96 Real-Time PCR system, and the QRT-PCR procedures were described as follows: pre-denaturation at 94 °C for 5 min, following 39 cycles for 30 s at 94 °C, annealing for 30 s at 58 °C and 30 s at 70 °C for extending. Gapdh was used as the loading control. Comparative CT values from QRT-PCR were used to measure relative gene expression. Primers are listed in Table S1.

### Immunocytochemistry

Cells were fixed in 4% paraformaldehyde in phosphate-buffered saline (PBS) at room temperature (RT) for 10 min, washed three times with PBS and then permeabilized for 15 min with 0.1% Triton-X 100 (Sigma Aldrich, St. Louis, MO) in PBS at RT. Cells were blocked with PBS supplemented with 4% bovine serum albumin (BSA) for 30 min, incubated with primary antibodies against CD61 (1:200; Abcam, UK), PRDM1 (1:400; Biolegend, USA), PRDM14 (1:500; Sigma), VASA, DAZL and PLZF (1:200; Abcam, UK) at 4 °C for 16 h. After washing with PBS for three times, cells were incubated with secondary antibodies for 1 h at 37 °C in the dark. Following another three washing steps in PBS, nuclei counterstaining was performed with 1 μg/ml Hoechst 33342 (Sigma Aldrich). Fluorescence images were obtained by Evos f1 fluorescence microscope (AMG, USA).

### Western blot

Total cell extracts were extracted in 1× sodium dodecyl sulfate polyacrylamide gel electrophoresis (SDS-PAGE) sample loading buffer. Total cell proteins were resolved by SDS-PAGE, transferred to Polyvinylidene difluoride membrane and probed with β-actin (1:1000, Beyotime, China), CD61 (1:200; Abcam), PRDM14 (1:500; Sigma), Sox2, CD49f, Stra8 (1:1000, Abcam) and Sycp3 (1:200, Beyotime). Horseradish peroxidase-conjugated anti-rabbit (1:1000, Beyotime) was used as a secondary antibody. Detection was performed using a Thermo Scientific Pierce enhanced chemiluminescence western blotting substrate (Thermo Scientific). Results were analysed by Tanon-410 automatic gel imaging system (Shanghai Tianneng Corporation, China).

### Statistical analysis

One-way analysis of variance (one-way ANOVA) was used and post-tests were conducted using Newman–Keuls multiple range test, if P-values were significant. Students’t-test was used when only two pairs of data were compared. All data were represented as mean ± SD, and statistical significance was expressed as follows: *P < 0.05; **P < 0.01; ***P < 0.001. All data were analysed using Graphpad Prism software (La Jolla, CA, USA) and were representative of at least three different experiments.

## Additional Information

**How to cite this article:** Fang, J. *et al*. CD61 promotes the differentiation of canine ADMSCs into PGC-like cells through modulation of TGF-β signaling. *Sci. Rep.* **7**, 43851; doi: 10.1038/srep43851 (2017).

**Publisher's note:** Springer Nature remains neutral with regard to jurisdictional claims in published maps and institutional affiliations.

## Supplementary Material

Supplementary Information
