# Supplementary material for: CD61 promotes the differentiation of canine ADMSCs into PGC-like cells through modulation of TGF-β signaling
Source: Sci Rep. 2017 Mar 3;7:43851. doi: 10.1038/srep43851 (PMC5335555; doi:10.1038/srep43851)

SUPPLEMENTARY INFORMATION

CD61 promotes the differentiation of canine ADMSCs into PGC-like cells through modulation of TGF-β signaling

Jia Fang[1], Yudong Wei[1], Changrong Lv[1], Sha Peng[1], Shanting Zhao[1*], Jinlian Hua[1*]

[1]College of Veterinary Medicine, Shaanxi Centre of Stem Cells Engineering & Technology, Northwest A&F University, Yangling, Shaanxi 712100, China

[*] **Corresponding author:** Jinlian Hua [E-mail:jinlianhua@nwsuaf.edu.cn]

Shanting Zhao [E-mail:zhaoshanting@nwsuaf.edu.cn]

SUPPLEMENTARY TABLE AND FIGURES

Table S1 Primers for QPCR

Gene	Strand	Sequence	PCR Product (bp)	Tm (℃)
Gapdh	F	GCTGCCAAATATGACGACATCA	71	60
	R	GTAGCCCAGGATGCCTTTGAG		
Sox2	F	CATGAACGGCTGGAGCAA	131	60
	R	GAGGAGGTAACCACGGGG		
Nanog	F	GATTCTTCCACCAGTCCGC	101	59
	R	CTCATCTTCTGTTTCTTGCCC		
Prdm1	F	GTGGTATTGTCGGGACTTTG	220	60
	R	AATGTTGGAGCGGTAGCA		
Prdm14	F	CTGTTACCCGAGGTGATGAA	376	59
	R	CTTTGGCAATGAGGCTACTG		
AP2γ	F	CCTGGGACTCCACGACAT	105	60
	R	GAAATAGGACCTTTGCGAATAA		
CD61	F	AGAGGAAGTGAAAAAGCAGAGTC	136	60
	R	GGTAAATACCAGCAAGTGGGA		
CD49f	F	CACGGATTGAATTTGACAACG	164	59
	R	ATCTCGGGATTCCTGCTTTG		
CD73	F	CGCTCAGAAAGTGAAGGGTGTG	133	60
	R	CCGCCCATCATCAGATGTGAC		

CD105	F	GGTTTCTGAGGGCTGCGTG	163	60
	R	CTTTGCACTGAGGACCAGGAAC		

Figure S1. Expression levels of meiotic markers in CD61-overexpressed cells, LY2109761-treated cells, TGF-β1-treated cells and control cells. The meiotic markers, STRA8 and SYCP3, were examined in cells treated with CD61-overexpression (a), LY2109761 (b) and TGF-β1 (c) by western blot. *$P<0.05$. **$P<0.01$.

Figure S2. Expressions of CD73 and CD105 in cADMSCs. The mRNA expressions of CD73 and CD105 in cADMSCs were detected by PCR.

Supplementary Figure 1

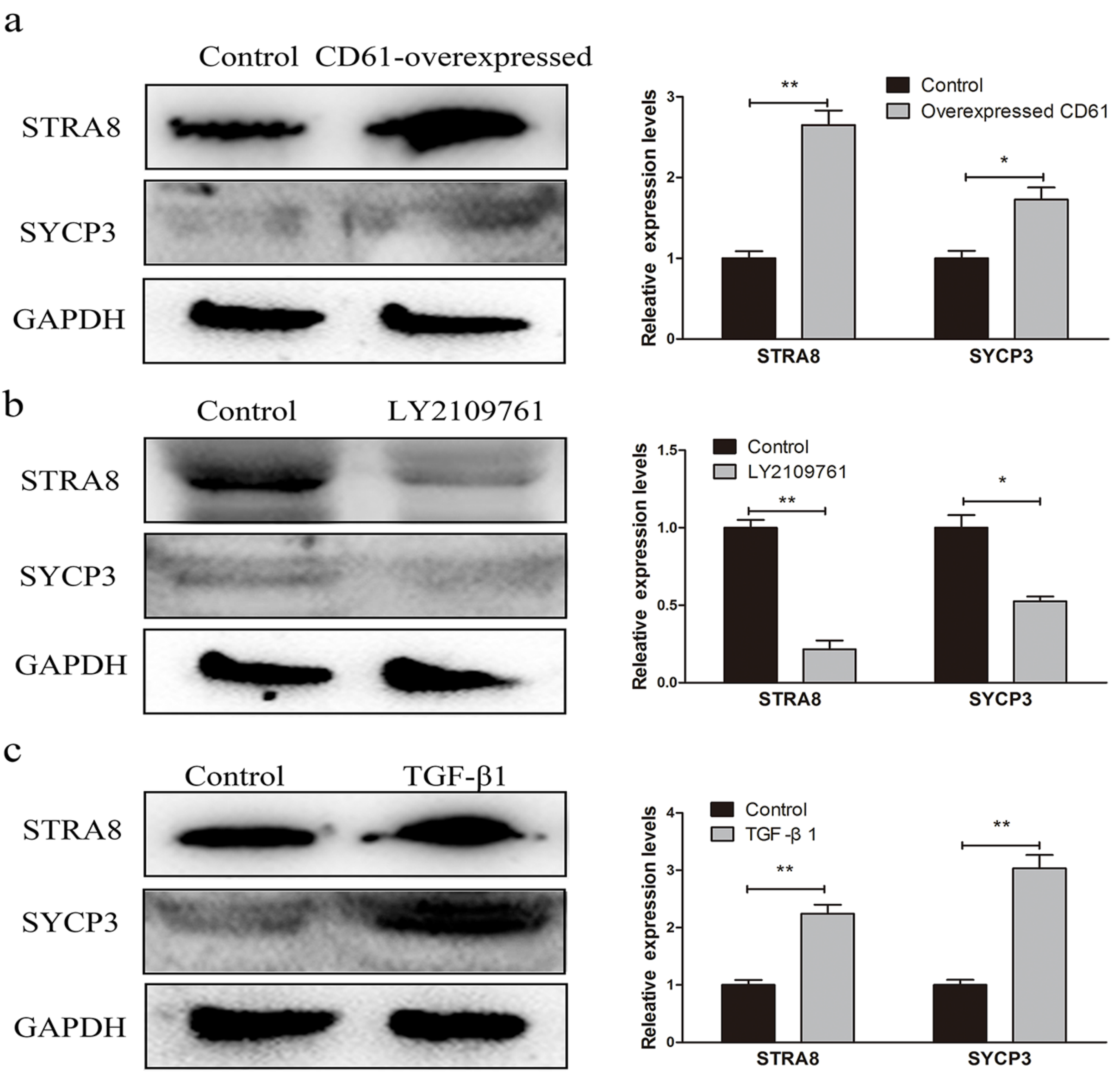

Supplementary Figure 2

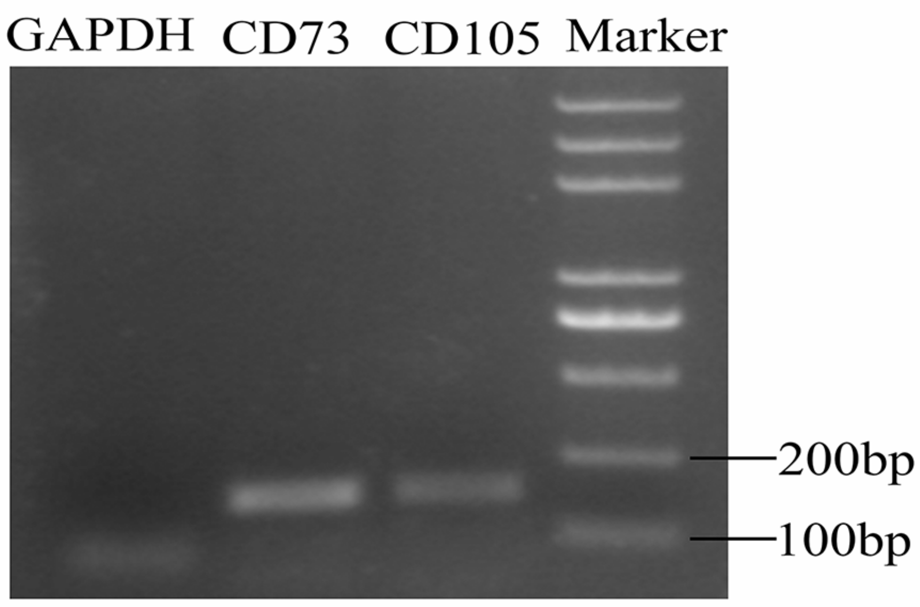

Supplement: Supplementary Information [file srep43851-s1.pdf]
